# Homopolymerization of Ethylene, 1-Hexene, Styrene and Copolymerization of Styrene With 1,3-Cyclohexadiene Using (η^5^-Tetramethylcyclopentadienyl)dimethylsilyl(N-Ar’)amido-TiCl_2_/MAO (Ar’=6-(2-(Diethylboryl)phenyl)pyrid-2-yl, Biphen-3-yl)

**DOI:** 10.3390/molecules16010567

**Published:** 2011-01-14

**Authors:** Sebnem Camadanli, Ulrich Decker, Christa Kühnel, Ingrid Reinhardt, Michael R. Buchmeiser

**Affiliations:** 1 Leibniz-Institut für Oberflächenmodifizierung e.V., Permoserstr. 15, D-04318, Leipzig, Germany; 2 Lehrstuhl für Makromolekulare Stoffe und Faserchemie, Institut für Polymerchemie, Universität Stuttgart, Pfaffenwaldring 55, D-70550, Stuttgart, Germany

**Keywords:** constrained geometry catalyst, olefin, homopolymerization, copolymerization

## Abstract

The propensity of a half-sandwich (η5-tetramethylcyclopentadienyl)dimethylsilylamido Ti^IV^-based catalyst bearing an auxiliary diethylboryl-protected pyridyl moiety (**Ti-8**), activated by methylaluminoxane (MAO) to homopolymerize α-olefins such as ethylene, 1-hexene and styrene as well as to copolymerize styrene with 1,3-cyclo-hexadiene is described. The reactivity of **Ti-8** was investigated in comparison to a 6-(2-(diethylboryl)phenyl)pyrid-2-yl-free analogue (**Ti-3**).

## 1. Introduction

**Figure 1:**
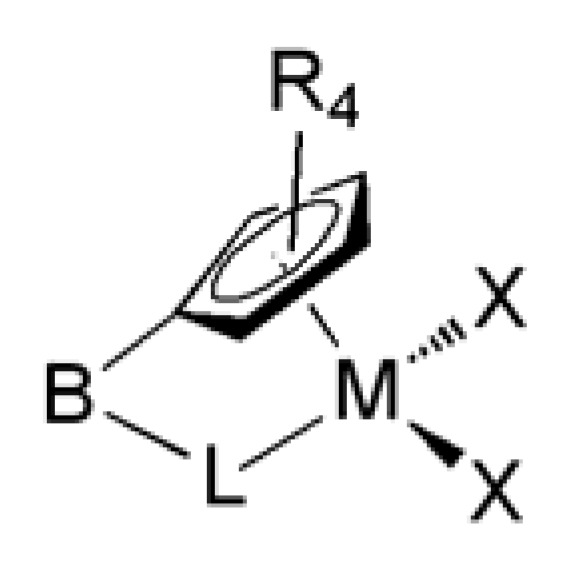
The constrained geometry catalyst (B: bridging unit, L: donating ligand and X: halide).

Polyolefins, the generic name for synthetic polymers based on ethylene, propylene and α-olefins, have become the world’s most common and the highest volume commercial class of synthetic polymers. The ability to make different kinds of materials, e.g., rigid thermoplastics, flexible elastomers or waxes from a very simple set of inexpensive building blocks is one reason for their broad utility [1]. There have been unparalleled developments in the design and production of single-site catalysts for the production of polyolefins over the past 20 years [2,3]. Most homogenous catalysts have been based on early transition metals [4]. The work by Sinn, Kaminsky, and Britzinger, demonstrating that Zr- and Ti-derived metallocenes combined with aluminoxanes lead to long-lived catalysts with very high activity, sparked the first industrial interest [5,6,7,8]. Important for the success of these systems is the formation of a cationic metal center, which is coordinatively unsaturated, highly electrophilic and, most important, stereochemically well defined. Metallocene analogues that have received much commercial attention are the group 4 constrained geometry catalysts (CGCs) developed concurrently by Dow and Exxon (Figure 1) [9,10]. Their key features are the open nature of the catalyst’s active site, which allows them to incorporate other olefins than ethylene, as well as their increased stability towards MAO as compared to metallocenes, which allows for higher polymerization temperatures and thus activities [11,12].

Exceptional catalytic activity of group IV CGCs for ethylene homopolymerization, the copolymerization of ethylene with α-olefins and the copolymerization of ethylene with styrene triggered intense research activity to understand, further improve and exploit the full catalytic potential of these compounds. We have prepared a catalytic system capable of synthesizing copolymers of ethylene with cyclic olefins such as norborn-2-ene and *cis*-cyclooctene that contain both ring-opening metathesis polymerization (ROMP) and vinyl insertion polymerization (VIP)-derived structures within one single polymer chain. 

**Figure 2:**
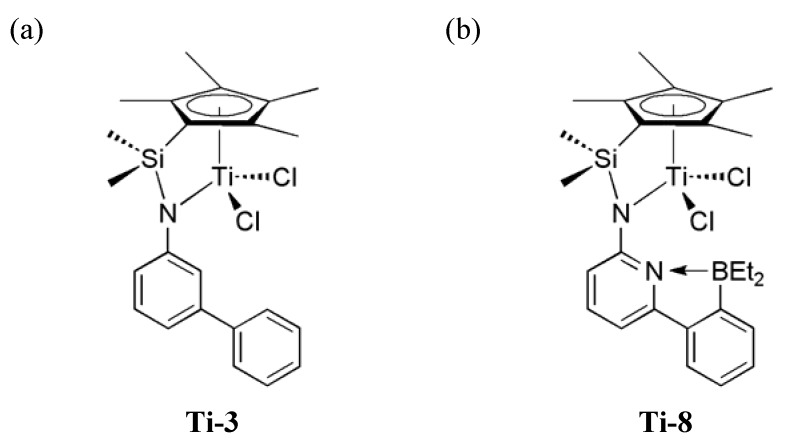
**(a)** (6-(2-(Diethylboryl)phenyl)pyrid-2-ylamido)(dimethylsilyl)(η^5^-tetramethyl-cyclopentadienyl)TiCl_2_: **Ti-8**; (b) (3-Biphen-3-ylamido)(dimethylsilyl)(η^5^-tetramethyl-cyclo-pentadienyl)TiCl_2_: **Ti-3**.

For such purpose, we designed a borylamido containing Ti^IV^-based, constrained geometry precatalyst (**Ti-8**) and compared its polymerization features to those of the corresponding borylamido-free model compound **Ti-3** (Figure 2) [13]. The ability of **Ti-8** to copolymerize cyclic olefins with ethylene both via vinyl insertion and ROMP is based on a reversible α-elimination step, which allows for the reversible creation of ROMP-active Ti-alkylidenes and their re-conversion into cationic, VIP-active Ti-complexes. Some zirconium and hafnium derivatives of **Ti-3**-type catalysts have also been synthesized by Voskoboynikov *et al.* and tested for ethylene and propylene homopolymerization [14].

**Scheme 1:**
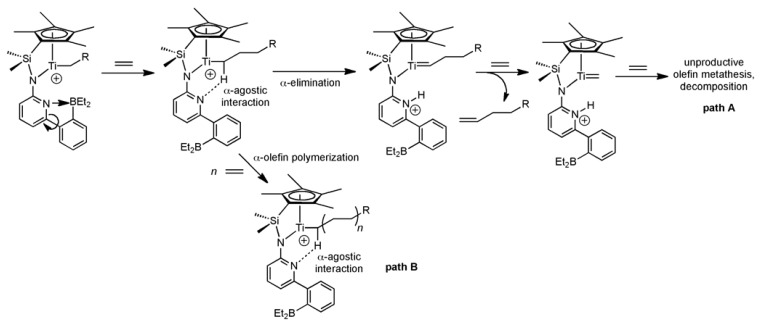
Chemistry of cationic half-sandwich catalysts capable of introducing α-elimination: vinyl insertion polymerization (VIP) *vs.* unproductive metathesis.

One crucial point in the copolymerization of an α-olefin, e.g., ethylene, styrene, or 1-hexene with a cyclic olefin such as norborn-2-ene, *cis*-cyclooctene or 1, 3-cyclohexadiene by a given catalyst is the propensity of this catalyst to *homopolymerize* the α-olefin of interest in the *absence* of a cyclic olefin. Such a homopolymerization (**path B**) is only possible if is not hampered by excessive, irreversible α-elimination, thereby resulting in the formation of metal alkylidenes (**path A**), which in turn give raise to unproductive olefin metathesis with ethylene. Thus, only in case the homopolymerization of ethylene or any 1-olefin can be accomplished, one can address their copolymerization in general and in particular copolymerizations with cyclic olefins under a reversible switch in the polymerization mechanism. In this contribution we report on that issue, *i.e.* on the homopolymerization of ethylene, styrene, 1-hexene and on the copolymerization of styrene with 1,3-cyclohexadiene with the **Ti-3**/MAO catalytic system in comparison to the **Ti-8**/MAO system. 

## 2. Results and Discussion

### 2.1. Synthesis and Homopolymerization of Ethylene

We already reported on the synthesis of the (η^5^-tetramethylcyclopentadienyl) dimethylsilylamido Ti^IV^-based catalysts **Ti-3** and **Ti-8** (Supporting Information) [13]. Ethylene polymerization using catalysts **Ti-8** and **Ti-3** was conducted in toluene in the presence of methylaluminumoxane (MAO). The results are summarized in Table 1. Generally, the properties of PE vary greatly depending on the polymer’s microstructure (*i.e.* linear or branched) from high-density plastics with relatively high melting points to low-density, branched materials, which are completely amorphous at room temperature. The mechanism by which ethylene polymerizes will ultimately determine the microstructure and therefore the properties of the resultant PE [15]. 

**Table 1:** Homopolymerization of ethylene using **Ti-8** and **Ti-3**.

#	Ti-8:MAO	T (°C)	Polymer yield (g)	Activity^[a]^	*M_n _*(g/mol)^[b]^	PDI	*T_m_* (°C)
1	1:1000	50	1.03	110	1,800,000	1.02	131
2	1:1000	75	1.83	195	1,300,000	1.21	132
**#**	**Ti-3:MAO**	**T (°C)**	**Polymer yield (g)**	**Activity^[a]^**	***M_n_* (g/mol)^[b]^**	**PDI**	***T_m_* (°C)**
3	1:1000	50	2.87	270	1,500,000	1.14	134

Reaction time: 1 h. Ethylene pressure: 4 bar. Amount of catalyst: 5 mg; [a] kg/mol^.^bar^.^h. [b] determined by high-temperature GPC in 1,2,4-trichlorobenzene.

**Figure 3:**
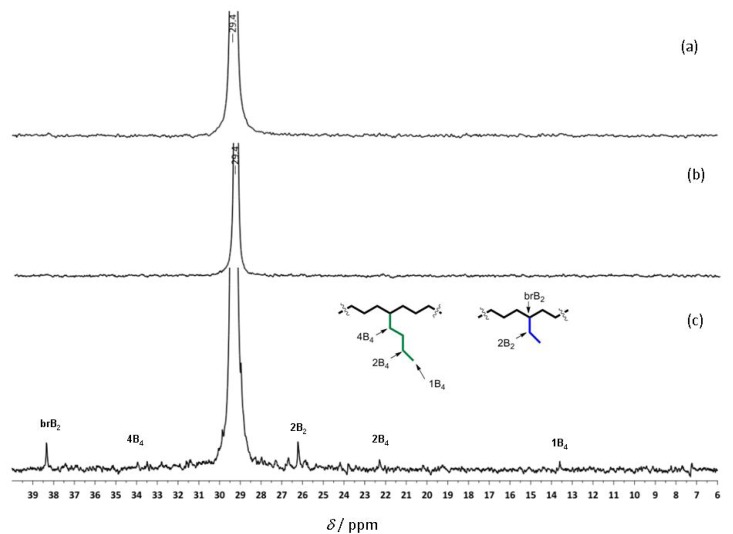
^13^C-NMRspectra of PE obtained by the action of **(a) Ti-3** at 50 °C, **(b) Ti-8** at 50 °C, **(c) Ti-8** at 75°C (all activated by MAO).

Complexes **Ti-8** and **Ti-3** exhibited moderate activity in ethylene polymerization in the presence of MAO. The lower activity of **Ti-8** as compared to **Ti-3** is ascribed to the presence of the pyridylamido ligand. Thus, once α-elimination is induced, the resulting Ti-alkylidenes undergo metathesis with ethylene resulting in unstable Ti-methylidenes. In the presence of ethylene, these undergo non-productive olefin metathesis and decompose. The higher activity in ethylene polymerization of the model catalyst **Ti-3** at 50 °C as compared to **Ti-8**, however, can additionally be rationalized by the decrease in steric hindrance. The linear PE obtained with **Ti-8** possessed high molecular weights (1,300,000 < *M_n _*< 1,800,000 g/mol) with unimodal molecular distributions (1.02 < PDI < 1.20). The ^13^C NMR spectra of PE obtained by the action of **Ti-3**/MAO and **Ti-8**/MAO at 50 °C show one single peak at 29.4 ppm, which is assigned to the methylene units in linear polyethylene. As expected, **Ti-8** showed a higher activity at 75 °C than at 50 °C. As might be anticipated, the molecular weight of the polymer decreased with increasing temperature from 1,800,000 g/mol to 1,300,000 g/mol, e.g., because of an acceleration of the β-hydride chain-transfer reaction [16]. Consequently, the spectrum of PE obtained by the action of **Ti-8**/MAO at 75 °C show additional signals that can be assigned to branched structures (Figure 3). This suggests that at higher polymerization temperature, branched PE is formed by the *in situ* copolymerization of ethylene with α-olefin macromonomers produced through β-hydride elimination. The spectral signatures of isolated branches in linear PE have been assigned in great detail [17,18,19]. Here, the nomenclature of Usami and Takayama for isolated branches is used [20]. The practical limitations of the ^13^C-NMR technique, however, are significant. Thus, measuring the number of branches by ^13^C-NMR is inconvenienced by poor signal-to-noise ratios for some of the signals, even after prolonged acquisition times. In the ^13^C-NMR spectrum of PE prepared by the action of **Ti-8** at 75 °C, the presence of ethyl and butyl branches could be confirmed by assigning the resonances at *δ* = 26.2 and 38.4 ppm as well as at *δ* *=* 13.2 and 22.3 ppm, respectively. The signal at *δ* = 7.2 ppm can be assigned to the methyl group in the ethyl branch. 

The melting points (*T_m_*) of all polymers were in the range of 131–134 °C and thus in the expected *T_m_*-range typical of high-density PE (130–137 °C) [21].

### 2.3. Homopolymerization of 1-Hexene

**Scheme 2:**
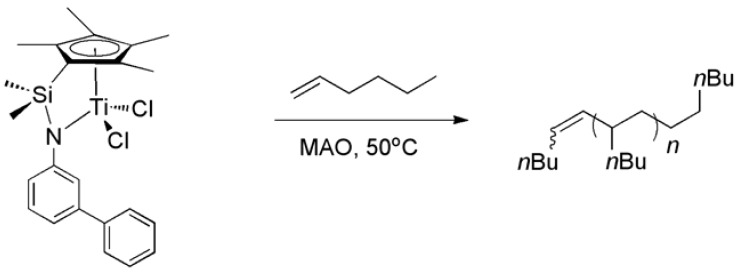
1-Hexene polymerization by the action of **Ti-3** activated by MAO.

Early-transition metal metallocene catalysts commonly incorporate α-olefins into the growing chain with 1,2-regioselectivity [22], whereas propagation with 2,1-regioselectivity [23,24] is mostly limited to non-metallocene systems. Okuda *et al*. reported that cationic group IV metal-based catalysts efficiently catalyze 1-hexene oligomerization and that the regioselectivity of insertion changes upon changing the metal center from titanium to zirconium or hafnium [25]. In the polymerization of 1-hexene with **Ti-3** activated by MAO at 50 °C, the regioselectivity switched during the reaction (Scheme 2), however, poly(1-hexene) instead of low-molecular weight oligomeric products (M_n _< 500 g/mol) [25] was obtained. The obtained poly(1-hexene) was a highly viscous liquid. It had a low molecular weight (M_n _= 25,000 g/mol) and a surprisingly low polydispersity (M_w_/M_n _= 1.00) (Table 2).

**Table 2:** Homopolymerization of 1-hexene using **Ti-3**.

Ti-3:MAO:1-hexene	Activity^[a]^	Polymer yield (g)	*M_n_* ^[b]^ (g/mol)	PDI
1:1,000:10,000	86	0.18	25,000	1.00

Reaction time: 1 h. Reaction Temperature: 50 °C. Amount of catalyst: 1 mg; [a] kg/mol^.^bar^.^h. [b]determined by high-temperature GPC in 1,2,4-trichlorobenzene.

**Figure 4:**
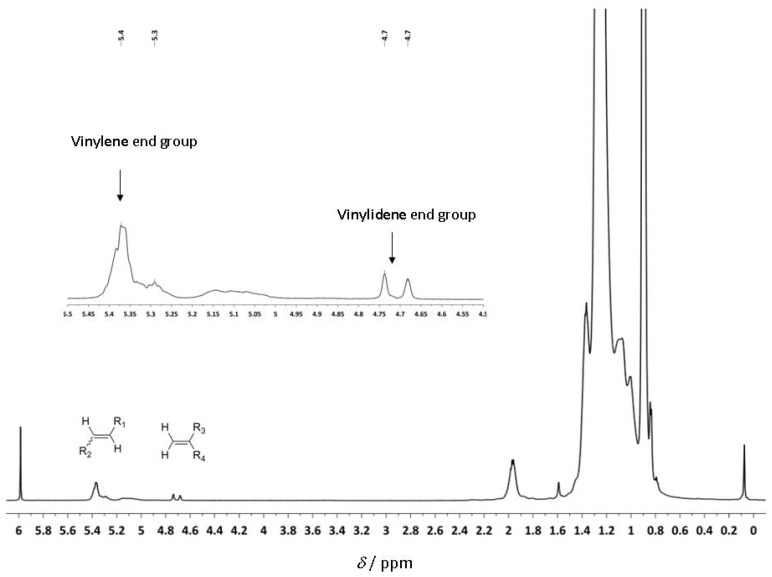
^1^H-NMR spectrum of poly(1-hexene) in C_2_D_2_Cl_4_.

**Figure 5:**
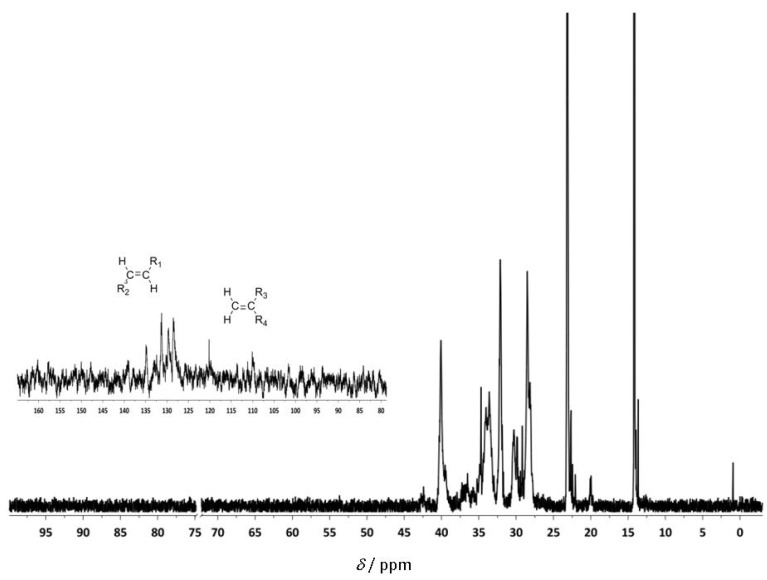
^13^C-NMR spectrum of poly(1-hexene) in C_2_D_2_Cl_4_.

The ^1^H-NMR spectrum shows olefin resonances at δ = 5.37 ppm, in agreement with the presence of vinylene groups [(E)- and (Z)-R^1^C*H*=C*H*R^2^] and at δ = 4.74 ppm signals for the vinylidene end groups (R^1^R^2^C=C*H*_2_) (Figure 4). The vinylene to vinylidene ratio was 98:2 (determined by ^1^H-NMR), which suggests that the polymerization was predominantly terminated via β-hydride elimination from a 2,1-enchained titanium alkyl complex. The ^13^C-NMR spectrum also supports the suggested mechanism (Figure 5). The signals around 130 ppm are assigned to the vinylene groups [(*E*)- and (*Z*)-R^1^*C*H=*C*HR^2^], those around 110 ppm to the vinylidene end group (R^1^R^2^*C*=*C*H_2_).

Although it is not possible to fully exclude an intermediary metallacycle, the cationic titanium alkyl species may account for the switch in regioselectivity due to solvent coordination. There, probably due to strong metal-solvent dissociation energies, the thermodynamically preferred 1,2-insertion then predominates. After initial 1,2-insertion into the metal-methyl bond, 1-hexene is enchained with 2,1-regioselectivity for titanium metal center. Larger growing chain and bulkier substrates on the ligand backbone cause steric hindrance. Therefore, dissociation of the solvent becomes more important. **Ti-8** is also capable of polymerizing 1-hexene, however, the observed activity was so low that we could not even obtain enough polymer for characterization. 

### 2.4. Homopolymerization of Styrene

**Scheme 3:**
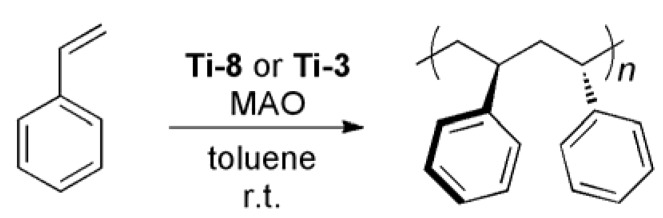
*st*-PS formation using **Ti-8** or **Ti-3**.

Syndiotactic polystyrene (*st*-PS) is an interesting class of polyolefins with unique properties that can only be prepared with homogenous olefin polymerization catalysts [26]. The syndiotactic (*st*) polymerization of styrene was first accomplished with Ti-based half-metallocenes in the presence of MAO by Ishihara and co-workers [27,28]. Since this initial report, *st*-PS has been subject of intense investigation because of its useful properties that include a high melting point (270 *°*C) and a low specific gravity, in addition to the general resistance to water and organic solvents at ambient temperature [29,30]. Catalysts **Ti-8** and **Ti-3** were also identified to efficiently polymerize styrene in a syndiospecific manner (Scheme 3). Chemical shifts are in accordance with published data [31,32,33].

**Figure 6:**
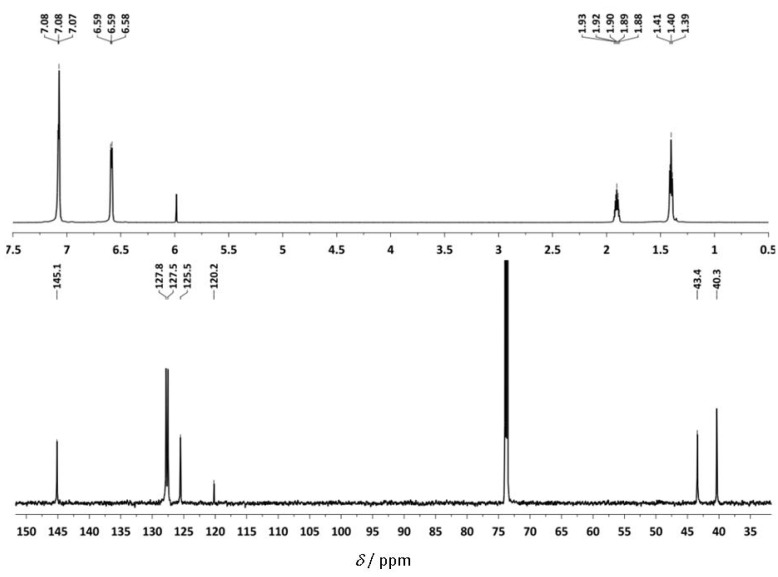
**(a)** ^1^H-NMR and **(b)** ^13^C-NMR spectra of st-PS by using **Ti-8** in C_2_D_2_Cl_4_.

The ^1^H-NMR and ^13^C-NMR spectra of st-PS are shown in Figure 6. Usually, the ^13^C-NMR signals of the phenyl ipso-carbons and the methylene carbons are used for determination of the tacticity of PS [34,35]. The high degree of stereoregularity causes each distinct carbon to appear as a single peak.

The presence of a sharp single signal for the quaternary carbon of the phenyl ring at *δ* = 145.1 ppm revealed that the polymer was highly *st*. Two sharp peaks at *δ* = 43.4 and 40.3 ppm were attributed to the main chain’s methyne and methylene carbon, respectively. The signal at *δ* = 120.2 ppm in ^13^C NMR stems from tetrachloroethane, an impurity from C_2_D_2_Cl_4_.

**Table 3:** Homopolymerization of styrene using **Ti-8** and **Ti-3**.

	Cat:MAO:Styrene	T (°C)	Activity ^[a]^	Yield (g)	*M_n_* ^[b] ^(g/mol)	PDI	*Tg/T_m_* (°C)
**Ti-8**	1:2000:10000	25	285	0.54	91,000	1.92	96/272
**Ti-3**	1:2000:10000	50	98	0.21	171,000	1.19	76/269

Reaction time: 1 h. Amount of catalyst: 1 mg; [a] kg/mol^.^bar^.^h. [b]determined by high-temperature GPC in 1,2,4-trichlorobenzene.

The polymerization results are summarized in Table 3. **Ti-8** polymerizes styrene with remarkable activity, however, the resulting polymer has a comparably low number-average molecular weight. While the PS produced by **Ti-3** is *st*, too, it has a number-average molecular weight twice as high as the polymer obtained with **Ti-8** and, most important, a low polydispersity index (PDI = 1.19). Therefore, it seems likely that the active species in these two systems is the same or very similar [36]. However, despite its higher molecular weight, *st*-PS produced by **Ti-3** possesses a lower *T_g_* (and comparable *T_m_*) than *st*-PS produced by **Ti-8**. This might stem from tiny amounts of atactic polystyrene (*at*-PS) in the *st*-PS produced by **Ti-3**. *at*-PS is miscible with *st*-PS [37], has a much lower *T_g_* than *st*-PS, and consequently reduces the overall *T_g_* value [38].

### 2.5. Homopolymerization of 1,3-Cyclohexadiene and its Copolymerization with Styrene

Poly(1,3-CHD) was successfully prepared by the action of **Ti-8** activated by MAO and shows a *T_g_* of 78 °C. The analysis of the microstructure with respect to the regioselectivity of insertion was performed according to the method of Williamson by comparing the integrations of the olefinic protons to the allylic protons in the ^1^H-NMR spectrum [39]. A ratio of approximately 40% of 1,2- *vs.* 60% of 1,4-addition was found. This is complemented by the ^13^C-NMR analysis, where a broad peak at δ = 130.8 ppm proves the 1,4-CHD unit, whereas intense signals between 128 < δ < 127 ppm are indicative or the 1,2-CHD unit [40,41]. (The signal at δ = 120.2 ppm in ^13^C-NMR stems from tetrachloroethane, an impurity in C_2_D_2_Cl_4_). 

**Scheme 4:**
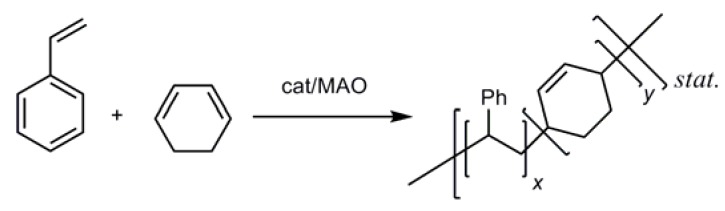
Copolymerization of styrene with 1,3-CHD.

Copolymers containing poly(1,3-cyclohexadiene) have recently attracted significant interest and a number of studies on the anionic copolymerization of styrene and 1,3-cyclohexadiene (1,3-CHD) [42] by an alkyl lithium initiator appeared [43,44,45,46,47]. These polymers show excellent thermal stability due to their high *T_g_*-values, which make them unique in the polydiene family. These unusual high *T_g_*-values were attributed to the cyclic structure of the repeat unit of poly(1,3-CHD), which obviously increases the degree of polymer rigidity relative to other acyclic dienes [48]. Here we report on the copolymerization of styrene with 1,3-CHD by the action of both **Ti-3** and **Ti-8** activated by MAO (Scheme 4). 

The results are summarized in Table 4. PS-*co*-poly(1,3-CHD) exhibit a higher *T_g_* (103 °C) than polystyrene. In fact the observed *T_g_* is similar to the ones found in copolymers prepared via the alternating anionic copolymerization of styrene and 1,3-cyclohexadiene [43]. Williamson *et al.* reported that *T_g_*-values were approximately 100 °C for all copolymer compositions. The presence of a single glass transition temperature also supports the formation of a single copolymer. Due to the very low ROMP-propensity of 1,3-cyclohexadiene, ROMP-derived structures can be ruled out. In addition, no additional signals, which could be assigned to such ROMP-derived structures can be seen, neither in the ^1^H- nor in the ^13^C-NMR spectra. 

**Table 4:** Copolymerization of styrene and 1,3-CHD using **Ti-8** and **Ti-3**.

	Cat:MAO:Styrene:1,3-CHD	χ_1,3-CHD_	Activity^[a]^	*M_n_* (g/mol)	PDI	*T_g_* (^o^C)	*T_m_* (^o^C)
**Ti-3**	1:2,000:10,000:10,000	0	170	117,000	1.45	86	271
**Ti-8**	1:2,000:10,000:10,000	>20^[b]^	640	288,000	1.89	103	-

Reaction time: 1h. T = 50 °C; [a] kg/mol^.^bar^.^h. n.d : not determined. χ_1,3-CHD_=mol-fraction of 1,3-CHD in the copolymer; ^[b]^ estimated from the ^13^C NMR.

**Figure 7:**
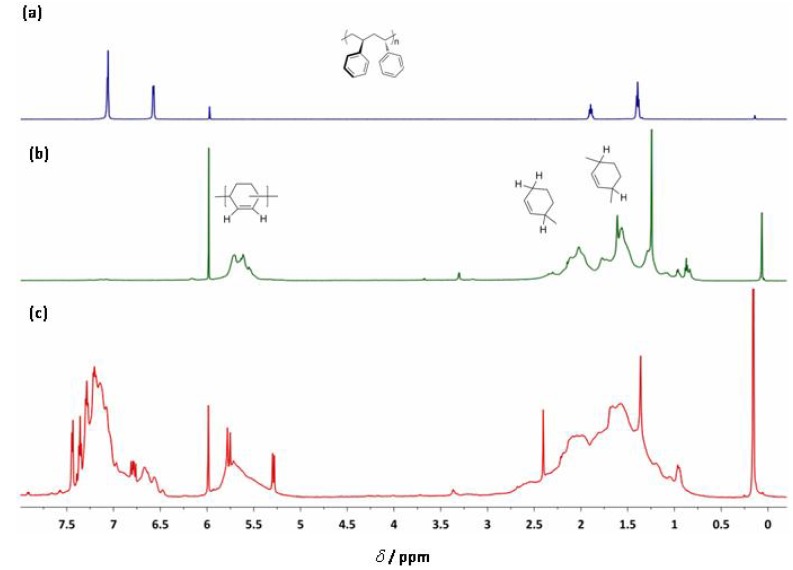
^1^H-NMR spectra of (a) PS (b) poly(1,3-CHD) (c) PS-*co*-poly(1,3-CHD) in CDCl_4_ prepared by the action of **Ti-8**/MAO.

**Figure 8:**
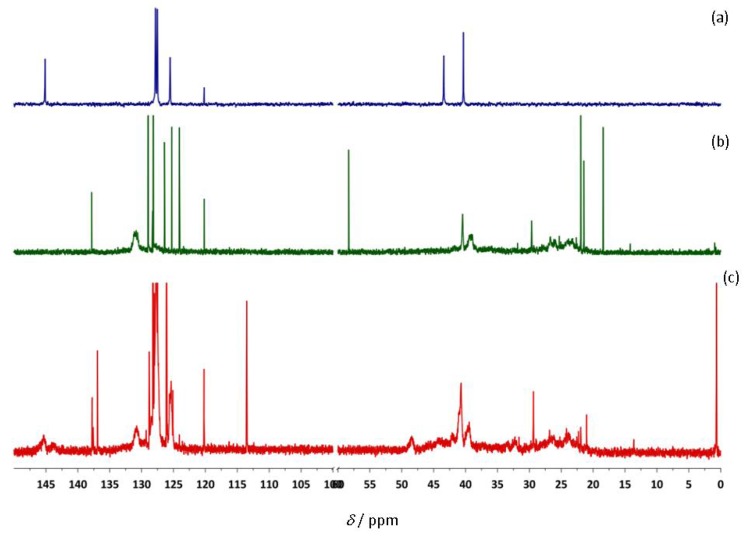
^13^C NMR spectra of (a) PS (b) poly(1,3-CHD) (c) PS-*co*-poly(1,3-CHD) in CDCl_4_ prepared by the action of **Ti-8**/MAO.

**Ti-3 **activated by MAO was *not* capable of copolymerizing styrene with 1,3-CHD. Only PS formed. However, we were able to prepare a high number-average molecular weight (*M_n _*= 288,000 g/mol) copolymer by using **Ti-8** activated by MAO. The resulting polymer was characterized by both ^1^H- (Figure 7) and ^13^C-NMR (Figure 8). 

The microstructure of the copolymer of 1,3-CHD with styrene can at least be elucidated though not perfectly analyzed via both ^1^H- and ^13^C-NMR. In the ^1^H-NMR, both the signals of PS and poly(1,3-CHE) can be found, however, additional signals of a terminal vinyl group at δ = 5.29 (d, *J* = 10.8 Hz), 5.78 (d, *J* = 17.5 Hz) and 6.79–6.97 ppm (dd, *J_1 _*= 17 Hz, *J_2 _*= 11 Hz) become visible and can, together with the signals around δ = 7.4 ppm be attributed to non-reacted, free styrene, which could hardly be removed from the copolymer. In addition, more olefinic and aromatic signals appear in the region 7.5 < δ < 6.5 ppm than could be expected for a simple block copolymer, i.e. PS-b-poly(1,3-CHD). In the ^13^C-NMR, the signals for st-PS, though very broad, appear at δ = 145 ppm. Also, the signals for poly(1,3-CHD) in the range 131 < δ < 125 ppm can be found. The sharp signals at δ = 113.5, 126.1, 128.0, 128.8, 136.9 and 137.6 ppm stem from free styrene. However, as in the ^1^H-NMR, additional signals in the region 131 < δ < 125 ppm can be observed. In view of these additional signals in both the ^1^H- and ^13^C-NMR, a random copolymer structure must be assumed for PS-co-poly(1,3-CHD). Unfortunately, because of these additional signals, the overall composition of the copolymer, i.e. the relative ratio of PS vs. poly(1,3-CHD) units cannot be accurately calculated from the integrals for the phenyl group of styrene (7.5 < δ < 6.5 ppm) and the olefinic signals of the poly(1,3-cyclohexadiene) units (5.8 < δ < 5.3 ppm). Nevertheless, a comparison of the signals at δ = 40.3 ppm (CH of PS) with the signals around 41 < δ < 39.5 ppm (CH-groups of poly(1,3-CHD)) allows for a rough estimation of that least 20 mol-% of PS being present within the copolymer. 

**Figure 9:**
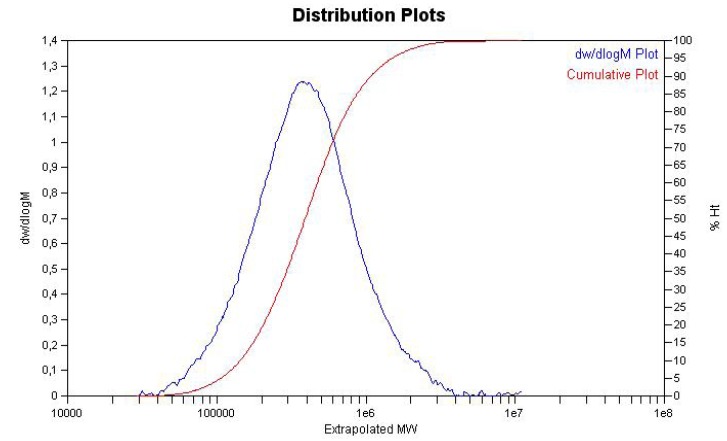
GPC-trace of PS-*co*-poly(1,3-CHD) prepared by the action of **Ti-8**/MAO.

The monomodal character of the resulting polymer (Figure 9), its comparably low PDI (1.89) as well as the NMR spectra strongly suggest the formation of a copolymer, *i.e.* PS-*co*-poly(1,3-CHD) instead of a mixtures of two homopolymers, *i.e.* of poly(1,3-CHD) and PS. 

## 3. Experimental

### 3.1. General

Methylalumoxane (MAO, 10 wt.-% solution in toluene) was purchased from Aldrich. The toluene was removed *in vacuo* and the remaining white powder was dried *in vacuo* to remove any free trimethylaluminium. Ethylene gas was purchased from Air Products and purified by passing through columns filled with BASF catalyst R 3-11G (Ludwigshafen, Germany) and 3 Å molecular sieves (Aldrich). All homopolymerization reactions were performed in Schlenk tubes under an inert atmosphere. All copolymerization reactions of ethylene with cyclic olefins were performed in a Buchi-Uster Pressure Reactor (polyclave) equipped with a Huber Thermostat (Unistat Tango Nuevo). The monomer feed of the gaseous monomer was kept constant with a Büchi pressflow bpc 6010 flow controller. The reaction was monitored by a bdsmc Büchi data system. NMR spectra were recorded on a Bruker Avance 600 (600.25 MHz for proton and 150.93 MHz for carbon) spectrometer at 20 °C unless specified otherwise. Proton and carbon spectra were referenced to internal solvent resonances and are reported in ppm. Molecular weights and molecular weight distributions were measured by high temperature gel permeation chromatography (HT-GPC) on three consecutive Waters Styragel HR4 4.6 × 300 mm columns in 1,2,4-trichlorobenzene at 145 °C using a 1515 isocratic pump, a 1717 Autosampler and a 2415 refractive index (RI) detector (all Waters Co, USA). The flow rate was set to 1 mL/min. Narrow PS standards in the range 162 < *M_n _*< 6035000 g^.^mol^-1^ (Easi Vial-red, yellow and green) were purchased from Polymer Labs. DSC data were recorded by heating under a nitrogen atmosphere on a DSC7 Perkin-Elmer differential scanning calorimeter. The glass transition temperature (*T_g_*) of the polymers was determined by TMA using a UIP-70 M thermomechanical analyzer at a heating rate of 2.5 °C/min. 

### 3.2. Homopolymerization of Ethylene

Polymerizations were carried out in a 500 mL Büchi autoclave reactor. The reactor was dried for 4 hrs under vacuum at 100 °C, cooled to room temperature, and flushed several times with argon. The solvents and reactants (250 mL of toluene, MAO and a toluene solution of the catalyst) were prepared in a glove-box and then introduced into the reactor under an argon atmosphere. The solution was then kept under constant stirring at 400 rpm and heated to the polymerization temperature (50 °C or 75 °C), and the ethylene pressure was raised to 4 bar. The ethylene pressure and reactor temperature were monitored and kept constant throughout the polymerization. The consumption of ethylene was followed continuously using a mass flow controller connected to a computer. The polymerization was stopped by closing the monomer supply and injecting methanol to the reactor. The polymer product was then poured into 500 mL of 10% HCl/methanol mixture, stirred for an hour, filtered, washed with water, and dried under vacuum for 24 h at 50 °C. 

### 3.3. Homopolymerization of Styrene and 1-Hexene

A 25 mL Schlenk flask equipped with a magnetic stirrer, was evacuated *in vacuo* and then filled with monomer, then the appropriate volume of catalyst solution (in toluene) and co-catalyst were injected. Polymerizations were carried out for 1 h at room temperature for styrene with **Ti-8** and at 50 °C for styrene with **Ti-3** and 1-hexene, respectively, and finally quenched with 10% HCl in methanol (200 mL). The precipitated polymer was filtered, washed with water and then dried overnight *in vacuo*. 

### 3.4. Copolymerization of Styrene with 1,3-Cyclohexadiene

A 20 mL Schlenk flask equipped with a magnetic stirrer was evacuated *in vacuo* and then filled with equimolar amounts of styrene and 1,3-cyclohexadiene, respectively. Then the appropriate amounts of catalyst and co-catalyst were combined in a beaker and injected into the system. The polymerization was carried out for 1 h at room temperature and then quenched with 10% HCl in methanol (50 mL). The precipitated polymer was filtered, washed with water and then dried overnight in a vacuum oven at 50 °C.

## 4. Conclusions

In summary, two constrained geometry-type catalysts, **Ti-8** and **Ti-3**, exhibited activity in ethylene homopolymerization and produced high molecular weight linear PEs with narrow polydispersity indices. **Ti-3** was effective in the polymerization of 1-hexene, where a switch in regioselectivity was observed during the polymerization. Both catalysts were also effective in the homopolymerization of styrene, though to a different extent, and *st*-PS with a high degree of stereoregularity was obtained. **Ti-3** activated by MAO also copolymerizes styrene with 1,3-cyclohexadiene resulting in copolymers with a comparably high poly(1,3-cyclohexadiene) content. 
